# Crystal structure of a short-chain dehydrogenase/reductase from *Burkholderia phymatum* in complex with NAD

**DOI:** 10.1107/S2053230X22000218

**Published:** 2022-01-27

**Authors:** Jawaher Alenazi, Stephen Mayclin, Sandhya Subramanian, Peter J. Myler, Oluwatoyin A. Asojo

**Affiliations:** a Department of Chemistry and Biochemistry, Hampton University, 200 William R. Harvey Way, Hampton, VA 23668, USA; b UCB Pharma, Bedford, Massachusetts, USA; c Seattle Structural Genomics Center for Infectious Disease (SSGCID), Seattle, Washington, USA; d Center for Global Infectious Disease Research, Seattle Children’s Research Institute, 307 Westlake Avenue North Suite 500, Seattle, Washington, USA

**Keywords:** SSGCID, oxidoreductases, *Burkholderia phymatum*, short-chain dehydrogenase/reductase family, NAD, structural genomics, Seattle Structural Genomics Center for Infectious Disease

## Abstract

*Burkholderia phymatum* is an important symbiotic nitrogen-fixing betaproteobacterium. *B. phymatum* is beneficial, unlike other *Burkholderia* species, which cause disease or are potential bioagents. Structural genomics studies at the SSGCID include characterization of the structures of short-chain dehydrogenases/reductases (SDRs) from multiple *Burkholderia* species. The crystal structure of a short-chain dehydrogenase from *B. phymatum* (*Bp*SDR) was determined in space group *C*222_1_ at a resolution of 1.80 Å. *Bp*SDR shares less than 38% sequence identity with any known structure. The monomer is a prototypical SDR with a well conserved cofactor-binding domain despite its low sequence identity. The substrate-binding cavity is unique and offers insights into possible functions and likely inhibitors of the enzymatic functions of *Bp*SDR.

## Abstract 2

The crystal structure of a short-chain dehydrogenase/reductase from *Burkholderia phymatum* offers insights into its possible functions and likely inhibitors of its enzymatic functions.

## 1. Introduction

*Burkholderia* are nonfermenting motile Gram-negative bacteria that are among the largest groups of species of Betaproteobacteria and include infective and symbiotic species (Yabuuchi *et al.*, 1992 ▸; Sawana *et al.*, 2014 ▸). *Burkholderia* can cause serious infections in humans; for example, *B. pseudomallei* causes melioidosis, a deadly emerging opportunistic infection (Hall *et al.*, 2019 ▸; Poe *et al.*, 1971 ▸; Veluthat *et al.*, 2021 ▸), and *B. glumae* and *B. gladioli* cause infections in plants (Zhou-qi *et al.*, 2016 ▸). *B. phymatum* was identified in root-nodule isolates from tropical legumes and is capable of symbiotic nitrogen fixation with the legume *Machaerium lunatum* and *Mimosa pudica* (Vandamme *et al.*, 2002 ▸). Due to the importance of *Burkholderia*, there is a need to clarify the structures of enzymes that play important roles in the life cycles of these bacteria. The SSGCID has characterized the structures of proteins that may play important roles in these bacteria. The short-chain dehydrogenase/reductases (SDRs) are NAD(P)(H)-dependent oxido­reductases that may be involved in the metabolism of diverse molecules, including lipids, amino acids, carbohydrates, cofactors, hormones and xenobiotic or other compounds. The protein structure reported here is a putative SDR which shares less than 38% sequence identity with any published structure. This structure is part of structural genomics efforts at the Seattle Structural Genomics Center for Infectious Disease (Raymond *et al.*, 2011 ▸; Myler *et al.*, 2009 ▸; Baugh *et al.*, 2013 ▸). This manuscript was completed in a partnership between Hampton University and the SSGCID (Asojo, Dranow *et al.*, 2018 ▸; Asojo, Subramanian *et al.*, 2018 ▸).

## 2. Materials and methods

### 2.1. Macromolecule production

SDR from *B. phymatum* (*Bp*SDR) was produced at the SSGCID (Myler *et al.*, 2009 ▸; Stacy *et al.*, 2011 ▸) following standard protocols described previously (Bryan *et al.*, 2011 ▸; Choi *et al.*, 2011 ▸; Serbzhinskiy *et al.*, 2015 ▸). Dr Mary Lidstrom of the University of Washington provided genomic DNA from *B. phymatum* STM815 (Moulin *et al.*, 2014 ▸). The sequence (UniProt ID B2JGP2, GenBank ID ACC70227.1) encoding amino acids 1–289 was PCR-amplified from genomic DNA using the primers shown in Table 1 ▸. The resultant amplicon was cloned by ligation-independent cloning into pET-14b expression vector pBG1861 provided by Dr Wesley Van Voorhis of the University of Washington. The vector encodes a noncleavable hexahistidine fusion tag (MAHHHHHHM-ORF). The plasmid DNA was transformed into chemically competent *Escherichia coli* BL21(DE3) cells (Table 1 ▸).

Recombinant *Bp*SDR was purified by the standard two-step protocol consisting of immobilized metal-affinity chromatography (IMAC) followed by size-exclusion chromatography (SEC) at SSGCID. All chromatography runs were performed on an ÄKTApurifier 10 (GE) using automated IMAC and SEC programs as described previously (Bryan *et al.*, 2011 ▸). Specifically, SEC was performed on a HiLoad 26/600 Superdex 75 column (GE Healthcare) using a mobile phase consisting of 300 m*M* NaCl, 20 m*M* HEPES, 5% glycerol, 1 m*M* TCEP pH 7.0. *Bp*SDR eluted as a single peak with a projected molecular weight of 22 kDa, indicating that the protein could either be a monomer or a dimer in solution. Protein purity was assessed using a reduced SDS–PAGE gel. The peak fractions were concentrated to 44.8 mg ml^−1^ using an Amicon Ultra-15 30K molecular-weight cutoff concentrator (Millipore, Billerica, Massachusetts, USA). Aliquots of 200 µl were flash-frozen in liquid nitrogen and stored at −80°C until use for crystallization. The pure protein is available at https://apps.sbri.org/SSGCIDTargetStatus/Target/BuphA.00010.n, as are the expressing clones.

### 2.2. Crystallization

*Bp*SDR was crystallized using the sitting-drop vapor-diffusion method. Prior to crystallization, NAD was added to the protein stock to a final concentration of 4 m*M*. Crystallization drops consisting of 0.4 ml *Bp*SDR (45 mg ml^−1^) and 0.4 ml precipitant were equilibrated against 80 ml precipitant in the reservoir at 14°C. Crystals were obtained using condition C4 from the MCSG1 sparse-matrix screen [170 m*M* ammonium acetate, 85 m*M* sodium acetate–HCl pH 4.5, 25%(*w*/*v*) PEG 4000, 15%(*v*/*v*) glycerol] as the precipitant (Table 2 ▸). Upon harvesting, crystals were cryocooled by plunging them into liquid nitrogen without additional cryoprotection.

### 2.3. Data collection and processing

X-ray diffraction data were collected on LS-CAT beamline 21-ID-F at the Advanced Photon Source (APS). Diffraction data (Table 3 ▸) were integrated using *XDS* and reduced using *XSCALE* (Kabsch, 2010 ▸). Data quality was assessed using *POINTLESS* (Evans, 2006 ▸). Raw diffraction data images are available at https://proteindiffraction.org/project/5ig2/.

### 2.4. Structure solution and refinement

The *Bp*SDR structure was solved by molecular replacement (MR) using *MOLREP* (Lebedev *et al.*, 2008 ▸; Vagin & Teplyakov, 2010 ▸). The MR search model was the structure of a Rv0851c ortholog short-chain dehydrogenase from *Mycobacterium paratuberculosis* (PDB entry 3tjr; Baugh *et al.*, 2015 ▸), the structure with the closest amino-acid sequence identity to *Bp*SDR (38% at over 89% coverage). Model building and structure refinement were performed using iterative cycles of *Coot* (Emsley *et al.*, 2010 ▸) and *Phenix* (Liebschner *et al.*, 2019 ▸). The quality of the model was assessed using *MolProbity* (Chen *et al.*, 2010 ▸). Figures were prepared using *PyMOL* (DeLano, 2002 ▸). The resulting structure-refinement data are provided in Table 4 ▸.

## 3. Results and discussion

**Figure 1:**
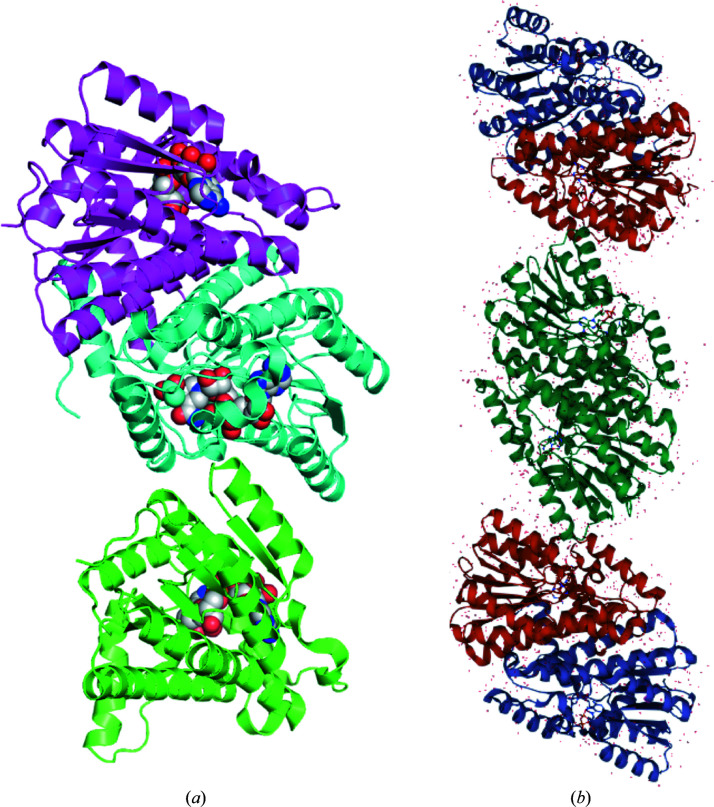
Crystal structure of *Bp*SDR. (*a*) A ribbon diagram of *Bp*SDR shows three monomers, colored magenta, cyan and aquamarine (NAD is shown as spheres), in the asymmetric unit. (*b*) Three prototypical SDR dimers can be generated from the *Bp*SDR structure (the red dots represent water molecules and ligands are shown as sticks).

**Figure 2:**
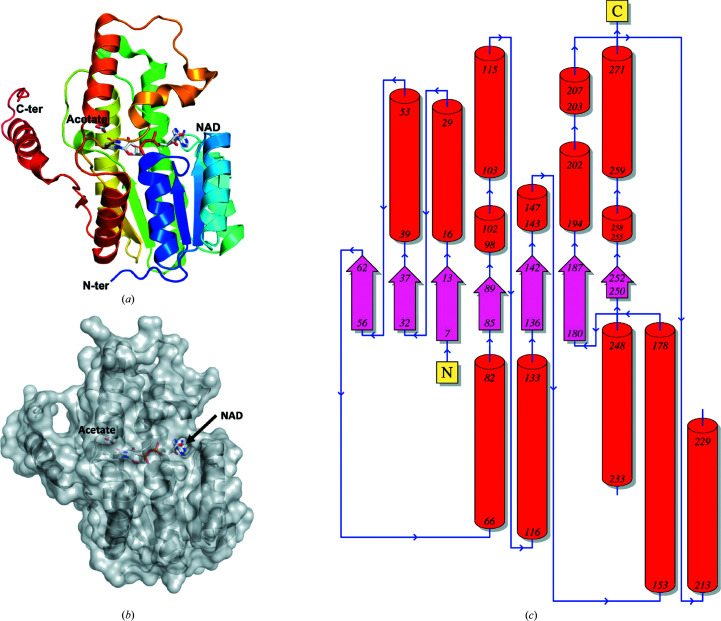
(*a*) Monomer structure of *Bp*SDR (PDB entry 5ig2) in a ternary complex with NAD and acetate. NAD binds in the cofactor-binding cavity, while acetate from the crystallization solution is in the substrate-binding site. The monomer is rainbow-colored from the N-terminus (blue) to the C-terminus (red). (*b*) Surface representation of the monomer structure of *Bp*SDR. (*b*) is in the same view as (*a*) and shows access tunnels to the cofactor-binding cavity. (*c*) Topology of *Bp*SDR. Helices are represented as cylinders, strands as arrows and loops as blue lines. The amino-terminus is labeled N and the carboxyl-terminus is labeled C.

The crystal structure of *Bp*SDR was solved in space group *C*222_1_ with three monomers in the asymmetric unit (Fig. 1 ▸). The three monomers of *Bp*SDR are very similar (with an r.m.s.d. of 0.119 Å for the alignment of all C^α^ atoms) and have the prototypical SDR topology (Fig. 1 ▸). Three dimers consistent with prototypical SDR dimers are easily generated by symmetry analysis (Fig. 1 ▸ *b*). The buried surface interface for each prototypical SDR dimer is large, including over 50 amino acids and covering ∼2640 Å^2^ per monomer. *Bp*SDR is a ternary complex, with each monomer having an acetate molecule in the substrate-binding domain and a NAD molecule in the cofactor-binding domain (Fig. 2 ▸ *a*). The cofactor-binding domain is the largest central cavity in the structure, and it is connected to the substrate-binding domain (Fig. 2 ▸ *b*). The 278 amino acid residues in each monomer have a secondary structure consisting of 12.2% β-strand, 47.8% α-helix and 6.5% 3_10_-helix and have the five β-α-β motifs of a prototypical SDR (Fig. 2 ▸ *c*).

**Figure 3:**
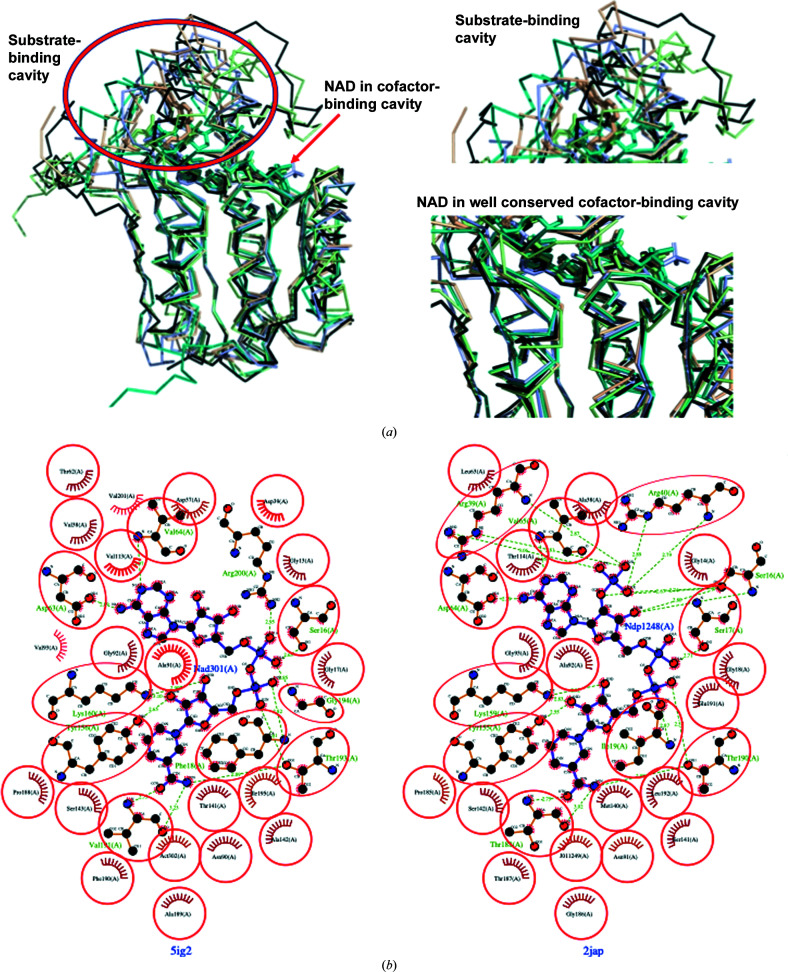
Comparison of representative SDRs with *Bp*SDR. (*a*) SDRs have a well conserved NAD-binding region and a variable substrate-binding region which determines their specificity. The PDB codes for the monomers are 6g41 (light orange), 2jap (light blue), 4yai (green), 1fmc (teal) and 5ig2 (black). (*b*) Side-by-side comparison of the NAD-binding domains of *Bp*SDR (PDB entry 5ig2) with its closest structural neighbor the Rv0851c ortholog short-chain dehydrogenase from *M. paratuberculosis* (PDB entry 3tjr) reveal conserved residues involved in hydrogen bonds (dashed green lines); the hydrophobic interactions that predominate in the NAD-binding domain are shown as red arcs with lines. Corresponding atoms that are involved in hydrophobic contacts are shown in red circles.

The most similar structures to *Bp*SDR were identified using *PDBeFold* analysis (http://www.ebi.ac.uk/msd-srv/ssm) at the default threshold cutoff of 70%. *PDBeFold* analysis revealed that the MR search model (PDB entry 3tjr; Baugh *et al.*, 2015 ▸) has the highest structural similarity to *Bp*SDR. To compare the substrate-binding cavities and determine whether the small molecules that bind to similar SDRs can bind to *Bp*SDR, the closest structures with bound ligands in their substrate-binding domain were selected from the results of *PDBeFold* analysis. The inhibitor-bound structures identified from this analysis include PDB entry 1fmc (7α-hydroxysteroid dehydrogenase in complex with NADH and 7-oxoglycochenodeoxycholic acid; Tanaka *et al.*, 1996 ▸), PDB entry 4yai (Ligl from *Sphingobium* sp. strain SYK-6 in complex with NADH and GGE; Pereira *et al.*, 2016 ▸), PDB entry 2jap (clavulanic acid dehydrogenase; MacKenzie *et al.*, 2007 ▸), PDB entry 6g4l (17β-hydroxysteroid dehydrogenase type 14 mutant Y253A in complex with a non­steroidal inhibitor; Badran *et al.*, 2019 ▸) and PDB entry 3tjr (crystal structure of the Rv0851c ortholog short-chain dehydrogenase from *M. paratuberculosis*; Baugh *et al.*, 2015 ▸). The superposed structures reveal conserved cofactor-binding domains and varied substrate-binding cavities (Figs. 3 ▸ and 4 ▸).

**Figure 4:**
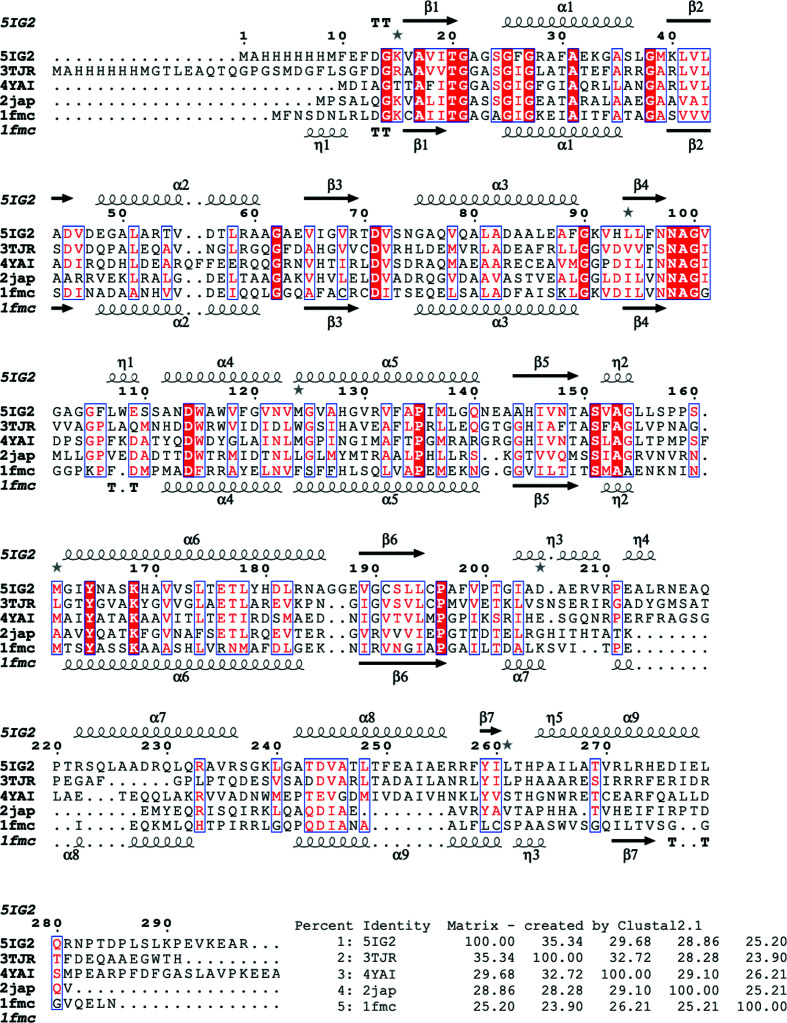
Structural and primary-sequence alignment of SDRs. The SDRs that are compared with *Bp*SDR (PDB entry 5ig2) are the Rv0851c ortholog short-chain dehydrogenase from *M. paratuberculosis* (PDB entry 3tjr), the 7α-hydroxysteroid dehydrogenase complex (PDB entry 1fmc), Ligl from *Sphingobium *sp. strain SYK-6 (PDB entry 4yai), clavulanic acid dehydrogenase (PDB entry 2jap) and 17β-hydroxysteroid dehydrogenase type 14 mutant Y253A (PDB entry 6g4l). The secondary-structure elements shown are α-helices (α), 3_10_-helices (η), β-strands (β) and β-turns (TT). Identical residues are shown in white on a red background and conserved residues are shown in red. This figure was generated using *ESPript* (Gouet *et al.*, 1999 ▸, 2003 ▸).

*LIGPLOT* analysis reveals a network of well conserved residues in the cofactor-binding domains (Fig. 3 ▸ *b*). *Bp*SDR has less than 40% sequence identity to other known homologs (Fig. 4 ▸). However, it maintains the same overall topology as found in other SDRs. Its cofactor-binding domain is virtually identical to that in *M. paratuberculosis* (PDB entry 3tjr). Some conformational flexibility is observed in the substrate-binding domain and the carboxyl-terminus (Fig. 4 ▸). The variability in the substrate-binding domain may be exploited for the design of selective inhibitors or for the development of novel biocatalysts for chiral transformations.

## 4. Conclusion

*Bp*SDR has the prototypical SDR topology, a well conserved cofactor-binding site and structural differences in the carboxyl-terminus and substrate-binding regions. Our ongoing studies include studying structural differences between SDRs in order to identify selective inhibitors for SDRs from bacterial pathogens closely related to *B. phymatum*.

## Supplementary Material

PDB reference: short-chain dehydrogenase/reductase from *Burkholderia phymatum* in complex with NAD, 5ig2

## Figures and Tables

**Table 1:** Macromolecule-production information

Source organism	*Burkholderia phymatum* (strain DSM 17167/STM815)
DNA source	Genomic DNA
Forward primer	5′-CTGCGAAAGCCGGAT-3′
Reverse primer	5′-TGCCATACTCTAGCYYGC-3′
Expression vector	pBG1861
Expression host	*E. coli* BL21 (DE3)
Complete amino-acid sequence of the construct produced	MAHHHHHHMFEFDGKVAVITGAGSGFGRAFAEKGASLGMKLVLADVDEGALARTVDTLRAAGAEVIGVRTDVSNGAQVQALADAALEAFGKVHLLFNNAGVGAGGFLWESSANDWAWVFGVNVMGVAHGVRVFAPIMLGQNEAAHIVNTASVAGLLSPPSMGIYNASKHAVVSLTETLYHDLRNAGGEVGCSLLCPAFVPTGIADAERVRPEALRNEAQPTRSQLAADRQLQRAVRSGKLGATDVATLTFEAIAERRFYILTHPAILATVRLRHEDIELQRNPTDPLSLKPEVKEAR

**Table 2:** Crystallization

Method	Vapor diffusion, sitting drop
Temperature (K)	287
Protein concentration (mg ml^−1^)	45
Buffer composition of protein solution	300 m*M* NaCl, 20 m*M* HEPES, 5% glycerol, 1 m*M* TCEP pH 7.0
Composition of reservoir solution	MCSG1 C4: 170 m*M* ammonium acetate, 85 m*M* sodium acetate–HCl pH 4.6, 25.5%(*w*/*v*) PEG 4000, 15%(*v*/*v*) glycerol, 4 m*M* NAD
Volume and ratio of drop	0.4 µl:0.4 µl

**Table 3:** Data collection and processing Values in parentheses are for the outer shell.

Diffraction source	APS beamline 21-ID-F
Wavelength (Å)	0.97872
Temperature (K)	100
Detector	RayoniX MX-225 CCD
Space group	*C*222_1_
*a*, *b*, *c* (Å)	83.52, 187.79, 108.26
α, β, γ (°)	90, 90, 90
Resolution range (Å)	46.895–1.800 (1.850–1.800)
Total No. of reflections	490706
Completeness (%)	99.900 (99.900)
Multiplicity	6.220 (6.23)
〈*I*/σ(*I*)〉	20.630 (3.69)
*R*_r.i.m._	0.071 (0.591)
Overall *B* factor from Wilson plot (Å^2^)	18.400

**Table 4:** Structure refinement Values in parentheses are for the outer shell.

Resolution range (Å)	46.8950–1.8000 (1.8530–1.8000)
Completeness (%)	99.9
σ Cutoff	*F* > 1.340σ(*F*)
No. of reflections, working set	77190 (6318)
No. of reflections, test set	1674 (128)
Final *R*_cryst_	0.141 (0.2382)
Final *R*_free_	0.177 (0.2715)
No. of non-H atoms	
Protein	6141
Ion	12
Ligand	132
Solvent	845
Total	7130
R.m.s. deviations	
Bonds (Å)	0.006
Angles (°)	0.777
Average *B* factors (Å^2^)	
Protein	21.6
Ion	47.4
Ligand	15.8
Water	33.7
Ramachandran plot	
Most favored (%)	99
Allowed (%)	1
